# Latent state-trait structure of BPRS subscales in clinical high-risk state and first episode psychosis

**DOI:** 10.1038/s41598-022-10207-x

**Published:** 2022-04-22

**Authors:** Lisa Hochstrasser, Erich Studerus, Anita Riecher-Rössler, Benno G. Schimmelmann, Martin Lambert, Undine E. Lang, Stefan Borgwardt, Rolf-Dieter Stieglitz, Christian G. Huber

**Affiliations:** 1 University Psychiatric Clinics Basel, University of Basel, Wilhelm Klein-Str. 27, 4012 Basel, Switzerland; 2 Klinik für Psychiatrie und Psychotherapie, Universitätsklinikum Hamburg-Eppendorf, Martinistr. 52, 20246 Hamburg, Germany; 3 University Hospital of Child and Adolescent Psychiatry, University of Bern, Effingerstr. 12, 3011 Bern, Switzerland; 4 Klinik für Kinder- und Jugendpsychiatrie, Universitätsklinikum Hamburg-Eppendorf, Martinistr. 52, 20246 Hamburg, Germany; 5 Department of Clinical Psychology and Psychiatry, University of Basel, Faculty of Psychology, Missionsstr. 60/62, 4055 Basel, Switzerland

**Keywords:** Psychiatric disorders, Psychosis

## Abstract

To investigate the longitudinal latent state-trait structure of the different dimensions of psychosis symptoms in clinical high-risk state (CHRS) and first episode psychosis (FEP) individuals over a one year time-span. This paper examines if the symptom clusters Positive Symptoms, Negative Symptoms, Affectivity, Resistance, Activation, and Excitement according to the Brief Psychiatric Rating Scale (BPRS) differ in their trait and state characters in 196 CHRS and 131 FEP individuals. Statistical analysis was performed using latent state-trait analysis. On average, trait differences accounted for 72.2% of Positive Symptoms, 81.1% of Negative Symptoms, 57.0% of Affectivity, and 69.2% of Activation, whereas 15.0% of the variance of Resistance and 13.2% of the variance of Excitement were explained by trait differences. Explorative analyses showed a trait components’ increase of 0.408 in Positive Symptoms from baseline up to the 9th month and an increase of 0.521 in Affectivity from baseline up to the 6th month. Negative Symptoms had the highest trait component levels of all subscales between baseline and 6 months. The finding that an increasing proportion of psychosis symptoms is persisting over time underlines the importance of early intervention programs in individuals with psychotic disorders.

## Introduction

Symptoms occurring in early stages of psychosis represent a heterogeneous psychopathological domain with symptom clusters ranging from hallucinations and thought disorder to emotional withdrawal, blunted affect, depression, and anxiety, and also including hostility, uncooperativeness, and excitement. This exemplary list underlines the diversity in the phenotype of psychotic disorders and hence supports the hypothesis of a multidimensional structure of psychotic symptomatology^1^. This applies not only to psychosis symptoms in the clinical population, but also to subclinical psychotic experiences in the general population^2^. Furthermore, previous research has shown that psychotic symptoms differ not only in their clinical expression but also in their longitudinal course: Van Os and colleagues for example showed that there is evidence for a psychosis continuum model ranging from subclinical psychotic experiences (observed experiences below the threshold of its clinical detection) in the general population to psychosis proneness/clinical high-risk state (describing people presenting with potentially prodromal symptoms^3,4^) and first episode psychosis called psychosis proneness-persistence-impairment model^5^. The majority of subclinical psychotic experiences seems to be episodic^5^. However, there is evidence, that these subclinical psychotic experiences (psychosis proneness) may become persistent and subsequently clinically relevant, depending on the degree of environmental exposure interacting with genetic risk. This approach is in line with the neurodevelopmental model of schizophrenia postulating that the mental disorder is the end state of abnormal neurodevelopmental processes that started years before the illness onset^6^. Rössler and colleagues also found that subclinical psychotic experiences are quite persistent in some individuals^7^. Another study of van Os and colleagues came to similar results: They found that subclinical psychotic experiences are transitory in about 80% of individuals, while around 20% go on to develop persistent psychotic experiences and 7% a psychotic disorder^8^. There is evidence that the clinical high-risk state is associated with an increased risk of psychotic disorders and its spectrum of related diagnostic categories such as schizophrenia, schizophreniform disorder, schizoaffective disorder, delusional disorder, brief psychotic disorder, depression/bipolar disorder with psychotic features, substance-induced psychotic disorder, psychotic disorder not otherwise classified^9,10^. A study of Rössler and colleagues differentiated between schizotypal signs (defined as the reduced capacity for close relationships as well as ideas of reference, odd beliefs, and suspicion/paranoid ideation) and schizophrenia nuclear symptoms (defined as thought insertion, thought-broadcasting, thought control, and hearing voices) and showed that the expression of these symptoms is predominantly influenced by stable traits around age 30, whereas the occasion-specific states are more influential at ages 20 and 50^11^. Thus, there might be clinical phenotypes with longitudinally more durable characteristics as well as occasion-specific states underlying psychoses.

The concept which these studies are based on is latent state-trait theory^12,13^, postulating that psychological constructs are not either a trait or a state but can be rather trait-like or rather state-like. Therefore, every characteristic comprises a state and a trait component but with differing proportions. According to the latent state-trait theory, a state represents systematic influences of the measurement situation (occasion-specific), whereas a trait represents temporally stable individual differences in the measured variables (consistent over occasions). A “situation” is defined as the bio-psycho-social conditions that are present in the measurements within a test occasion^14^. As an example of the differentiation between state and trait effects see Meyhöfer, Bertsch, Esser, and Ettinger (2016)^15^.

However, there are two questions that are unanswered at this point: (1) How is the proportion of occasion-specific states and stable traits in clinical high-risk state (CHRS) and first episode psychosis (FEP) individuals regarding psychosis symptoms? (2) Do the dimensions of psychotic psychopathology differ in their latent state-trait structure over time?

Our first hypothesis is that the dimensions of psychotic symptoms differ regarding their proportions of occasion-specific states and stable traits in CHRS and FEP individuals. Our second hypothesis is that the latent state-trait structure of the dimensions of psychotic psychopathology differs over time.

### Aim of study

Our aim was to determine the proportion of variance related to latent states and traits in CHRS and FEP individuals over a 1 year time-span.

## Methods

### Context and sample

This study presents a post-hoc analysis of data from CHRS individuals included in the longitudinal research program on the early detection of psychotic disorders (FePsy) at the University of Basel Psychiatric Hospital from April 2000 to May 2017, and of FEP individuals presenting at the Psychosis Early Detection and Intervention Centre (PEDIC) at the Department of Psychiatry, University Medical Centre Hamburg-Eppendorf (UKE Hamburg, Germany) from January 2005 to December 2008. The FePsy and the PEDIC are comparable ongoing and well-established programs for the prevention, early detection, and early treatment of psychosis and are reference centers for these individuals in their catchment areas^16–18^. With their long-term clinical programs and accompanying research, they address this area from an integrated point of view.

To be included, CHRS individuals had to meet the criteria for an at risk mental state in psychosis, namely: attenuated psychotic symptoms or brief limited intermittent psychotic symptoms according to the PACE criteria^19^, familial aggregation of psychotic disorders in combination with at least two further risk factors similar to the PACE criteria, or a minimal amount and combination of certain risk factors according to the Basel Screening Instrument for Psychosis^20^. Exclusion criteria were age < 18 years, insufficient knowledge of German, IQ below 85, previous episode of schizophrenic psychosis, psychosis due to organic reasons or substance abuse, or psychotic symptomatology within affective psychosis or borderline personality disorder. Individuals who were treated with antipsychotics > 3 weeks or who had exceeded a 2500 mg cumulative chlorpromazine equivalent dose were also excluded^21^.

Inclusion criteria for FEP individuals were age 14–65, a schizophrenia spectrum disorder diagnosis according to DSM-IV^22^, no psychotic episode in the past, and absence of organic disorders presenting with a psychotic syndrome and of mental retardation.

For a more detailed description of the in- and exclusion criteria see Riecher-Rössler et al. (2007 and 2009)^16,23^ and Hochstrasser et al. (2017)^24^.

Individuals received treatment as usual, ranging from active surveillance to integrated psychiatric treatment with antipsychotic pharmacotherapy as deemed necessary by the treatment team. Data were collected from service entry every three months over one year (study visits: baseline as well as 3-months, 6-months, 9-months and 12-months follow-up). CHRS individuals were followed-up for transition for a maximum of five years (data from year two to five were not considered in the present study). Once a CHRS individual transitioned, it was counted as dropout. Four individuals were excluded from the analysis because of not showing up for any of the study visits. The final sample consisted of 327 individuals, 196 CHRS and 131 FEP individuals.

Study procedures were conducted in accordance with all local and national regulations, and the local ethics committees (the Ethics Committee of Northwestern and Central Switzerland (EKNZ) and the Ethics Committee of the Ärztekammer Hamburg) approved the study protocols (2465/05, 2515/05, OB-026/06, M12/99). Informed consent has been obtained from each participant. For participants under the age of 18 years, informed consent has been obtained from a parent and/or legal guardian.

### Measures

Psychopathology was assessed by trained raters in the FePsy and PEDIC programs using the Brief Psychiatric Rating Scale^25^ (FePsy), and the Positive and Negative Syndrome Scale for Schizophrenia^26^ (PEDIC)^17,18,27^. The PANSS has been developed based on the BPRS, and therefore, all BPRS items can be derived from the corresponding PANSS items. In addition, there is broad evidence for the equivalence of the BPRS and the PANSS^28^. BPRS ratings for the PEDIC individuals could therefore be extracted from the PANSS ratings.

The BPRS subscales “Positive Symptoms” (items: unusual thought content, conceptual disorganization, hallucinatory behavior, and grandiosity), “Negative Symptoms” (items: blunted affect, emotional withdrawal, and motor retardation), “Affectivity” (items: anxiety, guilt feelings, depressive mood, and somatic concern), “Resistance” (items: hostility, uncooperativeness, and suspiciousness), and “Activation” (items: excitement, tension, and mannerisms-posturing) were calculated according to Shafer^29^. Furthermore, based on Huber et al. (2012), the BPRS Excited Component (BPRS-EC) (items: excitement, hostility, uncooperativeness, and tension) was calculated as an established measure of aggression and agitation constructed in accordance with the PANSS-EC^17,30–32^. As you can see here, there is a partial item overlap between the subscales Resistance, Activation and Excitement.

Concerning psychometric properties of the BPRS and the PANSS, Bell, et al. found high inter-rater reliabilities and good criterion validities^33^. The kappa coefficient of the PANSS total score in our data base is κ = 0.68. Kay, Opler and Lindenmayer (1988) found strong correlations of the PANSS and the Scale for the Assessment of Negative Symptoms (SANS) and the Assessment of Positive Symptoms (SAPS), which indicates a good construct validity^34^.

### Statistical analysis

Descriptive statistics are given in total numbers and percentages for nominal scaled variables as well as mean, standard deviation (*SD*), median (*MDN*), and range for ordinal and interval scaled variables.

**Figure 1:**
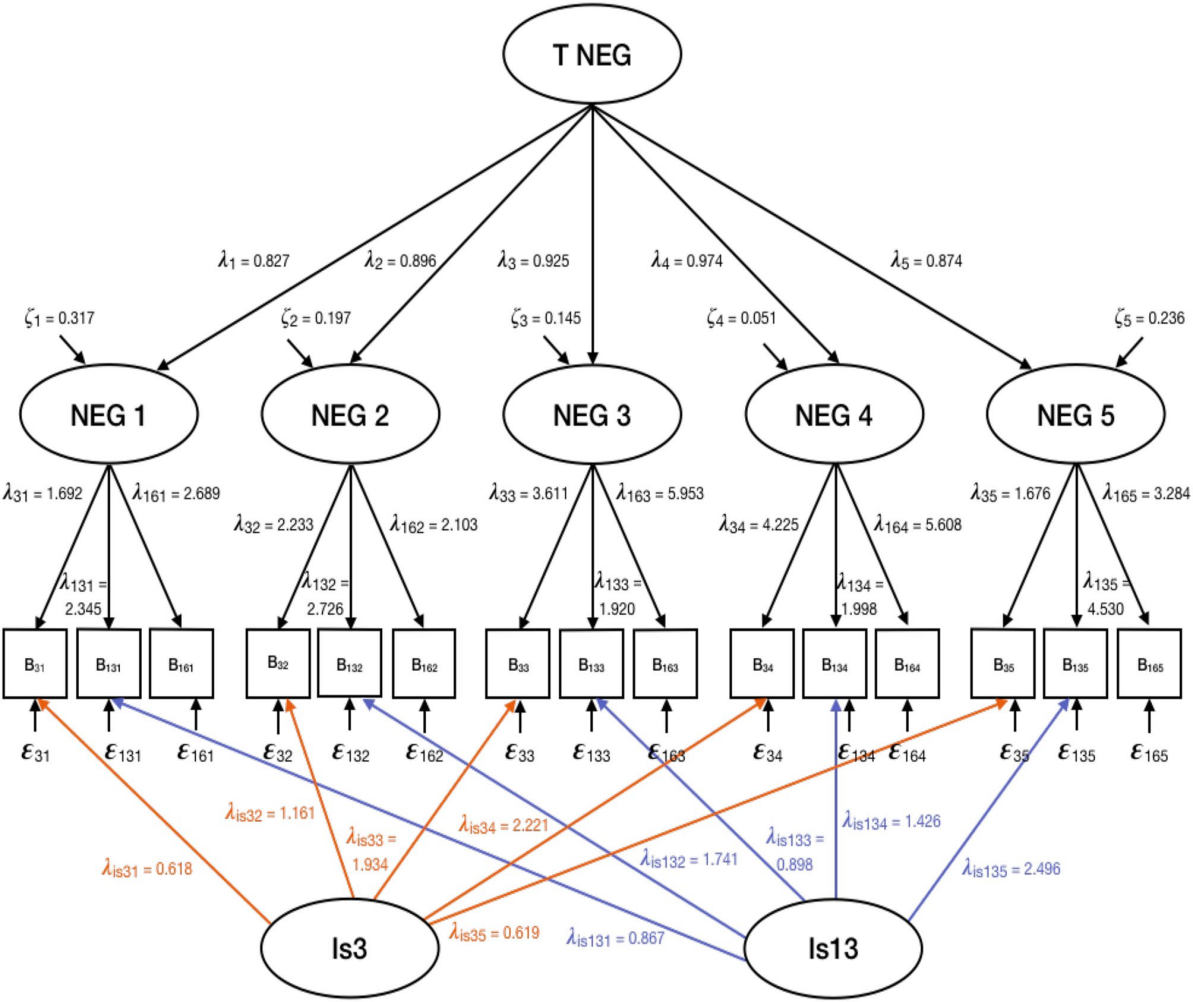
Latent state-trait model for Negative Symptoms (NEG). *T* common trait factor, *λ*_*k*_ factor loading parameters, *ζ*_*k*_ latent state residual variables, *B*_*ik*_ observed variables with *i* indicator and *k* measurement occasion, *ε*_*ik*_ error variables, *Is* indicator-specific trait factor.

The main analyses consisted of latent state-trait models decomposing the variance of the observed BPRS subscores into variance due to stable individual differences (trait component), variance due to the current study visit (state component), and error variance by using structural equation modeling^35^. Hence the latent state-trait model implies that the observed variables are affected by the individual and stable characteristics of the person, measurement errors and also situational factors as well as effects of the person-situation interaction^14^. For each symptom cluster, a latent state-trait model incorporating five lower order state factors (i.e. one for each study visit) and one higher order trait factor was fitted (see Fig. 1)^36^.

A consistency coefficient r^2^ was calculated for every subscale and each study visit indicating the proportions of variance explained by trait differences in BPRS subscales. As fit indices, the posterior-predictive *p*-value (*PPP*) and the difference between the observed and the replicated chi-square values (Δχ^2^) were used. *PPP*s as close as possible to 0.5 and positive upper levels of the *CI* indicate good fit^37^. In a first step, we performed the models without indicator-specific trait factors, but since these models did not fit the data, we included indicator-specific trait factors, which resulted in a good model fit. Indicator-specific trait factors (also called method-specific factors) are integrated in the latent state-trait model to take account of temporally stable components of the indicators not shared with the other indicators of the construct by allowing each indicator to load onto its own latent trait factor^38^.

Group differences (CHRS vs. FEP) regarding the extent of the trait component were directly tested within the structural equation modelling framework by regressing the group variable on the latent trait factor. To verify the assumptions of regression analyses we examined diagnostic residual plots, histograms and Q-Q-Plots. Furthermore, we controlled our analysis for the potential confounders age and gender by regressing these variables on the latent trait factor. All models were fitted in Mplus 7^39^ using Bayesian estimation and handled missing values using the Bayesian parametric multiple imputation approach, which is similar to the full information maximum likelihood estimation approach^40–43^.

In contrast to the frequentist inference approach, Bayesian analysis assumes that model parameters are random quantities, not constants, and thus can incorporate prior knowledge. Hence, Bayes combines prior distributions for parameters with the data likelihood to form posterior distributions for the parameter estimates. An important advantage of Bayesian analysis is the acquirement of more balanced results through using previous information mitigating the effect of a small sample size or/and many dropouts^44,45^.

### Ethics committee approval

Study procedures were conducted in accordance with all local and national regulations, and the local ethics committees (the Ethics Committee of Northwestern and Central Switzerland (EKNZ) and the Ethics Committee of the Ärztekammer Hamburg) approved the study protocols (2465/05, 2515/05, OB-026/06, M12/99).

## Results

### Descriptive statistics of the sample

Of the 327 individuals comprising the final study population, 175 (53.5%) were female and the mean age was 25.0 years (*SD:* 7.1, range: 14–57). 43 (21.9%) of the 196 CHRS individuals transitioned to first episode psychosis within the follow-up period for transition. Of the 131 FEP individuals, 53 (40.5%) individuals were diagnosed with schizophrenia, 37 (28.2%) with schizophreniform disorder, 12 (9.2%) with schizoaffective disorder, 11 (8.4%) with psychotic disorder not otherwise specified, 6 (4.6%) with delusional disorder and 12 (9.2%) with other schizophrenia-spectrum disorders. 96 (49.0%) of the 196 CHRS individuals and 124 (94.7%) of the 131 FEP individuals received medication (antipsychotics, antidepressants, anxiolytics, and/or mood stabilizers) at any time within the observation period.

### Missing data

147 (45.0%) of the participants could be retained over the whole study duration. To control for the effect of dropouts, the latent state-trait analysis was repeated for the subsample without dropouts and yielded qualitatively identical results. Additionally, we performed subgroup analyses for dropouts vs. non-dropouts comparing the sociodemographic variables gender, age, CHRS vs. FEP and BPRS at baseline. Gender, age and BPRS at baseline did not differ regarding dropout status, indicating that the data were missing at random concerning these variables. However, there were significantly more CHRS individuals (134; 74.4%) that dropped out during the observation period than FEP (46; 25.6%) individuals.

### Descriptive statistics of BPRS baseline scores

Descriptive statistics of BPRS baseline subscores for CHRS and FEP individuals are shown in Table 1.

**Table 1:** Descriptive statistics of BPRS baseline scores.

	POS		NEG		AFF		RES		ACT		EXC	
	CHRS	FEP	CHRS	FEP	CHRS	FEP	CHRS	FEP	CHRS	FEP	CHRS	FEP
M	6.0	11.6	5.4	9.8	9.3	13.6	4.8	8.2	4.1	7.7	5.6	10.1
SD	1.9	4.1	2.7	3.6	3.1	3.4	1.9	3.0	1.6	2.8	2.2	4.0
MDN	6	11	5	10	9	14	4	8	3	8	5	9
MIN	4	4	3	3	4	6	3	3	3	3	4	4
MAX	12	23	14	20	18	25	12	21	11	17	15	28
Skewness	1.14 (*SE* = 0.14)		0.62 (*SE* = 0.14)		0.31 (*SE* = 0.14)		1.28 (*SE* = 0.14)		1.15 (*SE* = 0.14)		1.48 (*SE* = 0.14)	
Kurtosis	0.89 (*SE* = 0.27)		−0.53 (*SE* = 0.27)		−0.25 (*SE* = 0.28)		2.34 (*SE* = 0.27)		1.33 (*SE* = 0.27)		3.06 (*SE* = 0.27)	
p-value	*p* < .001		*p* < .001		*p* < .001		*p* < .001		*p* < .001		*p* < .001	
Cohen’s d	1.77		1.38		1.32		1.39		1.61		1.39	
Cronbachs α	0.72		0.85		0.70		0.63		0.71		0.77	

*BPRS* Brief Psychiatric Rating Scale, *POS* Positive Symptoms (items: unusual thought content, conceptual disorganization, hallucinatory behavior, grandiosity; theoretical range: 4–28), *NEG* Negative Symptoms (items: blunted affect, emotional withdrawal, motor retardation; theoretical range: 3–21), *AFF* Affectivity (items: anxiety, guilt feelings, depressive mood, somatic concern; theoretical range: 4–28), *RES* Resistance (items: hostility, uncooperativeness, suspiciousness; theoretical range: 3–21), *ACT* Activation (items: excitement, tension, mannerisms-posturing; theoretical range: 3–21), *EXC* Excitement (items: excitement, hostility, uncooperativeness, tension; theoretical range: 4–28), *CHRS* Clinical High-Risk State subjects, *FEP* First Episode Psychosis individuals, *M* mean value, *SD* standard deviation, *MDN* median, *MIN* minimum, *MAX* maximum, *SE* standard error.

### Latent state-trait analyses

**Figure 2:**
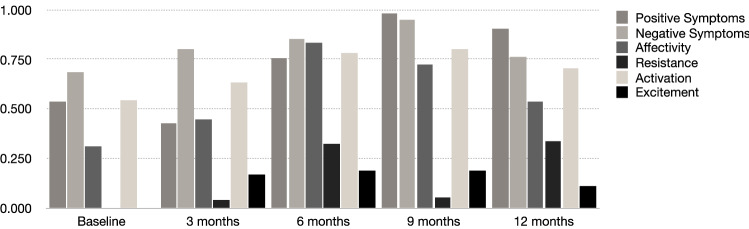
r^2^-values for BPRS subscales by study visit.

The results of the latent state-trait analyses showing the proportions of variance explained by trait differences (consistency coefficient r^2^) in BPRS subscales are presented in Table 2 and visualized in Fig. 2.

**Table 2:** Proportions of variance explained by trait differences (r^2^) in BPRS subscales.

	r^2^						Fit indices		
	Baseline	3 months	6 months	9 months	12 months	*M*	*PPP*	95% *CI* (Δχ^2^)	
Positive Symptoms	0.539	0.425	0.754	0.984	0.907	0.722	0.492	−56.863	66.668
Negative Symptoms	0.683	0.803	0.855	0.949	0.764	0.811	0.480	−45.123	44.989
Affectivity	0.311	0.446	0.832	0.725	0.534	0.570	0.384	−49.383	69.012
Resistance	0.000	0.041	0.321	0.052	0.334	0.150	0.472	−42.541	43.535
Activation	0.546	0.631	0.780	0.799	0.703	0.692	0.514	−48.670	45.371
Excitement	0.000	0.170	0.189	0.191	0.112	0.132	0.474	−57.679	62.703

*r*^*2*^ proportions of variance explained by trait differences (consistency coefficient), *BPRS* Brief Psychiatric Rating Scale, *M* mean value, *PPP* posterior predictive *p*-value (*PPP*s as close as possible to 0.5 indicate good fit), *Δχ*^*2*^ difference between the observed and the replicated chi-square values (positive upper levels of the *CI* indicate good fit).

On average, 57% to 81.1% of the variance of Positive Symptoms (72.2%), Negative Symptoms (81.1%), Affectivity (57.0%) and Activation (69.2%) across the five different time points was explained by trait differences. However, only 15.0% of the variance in Resistance and 13.2% of the variance in BPRS-EC was explained by trait differences across the five study visits.

### Explorative analyses

Explorative analyses showed a trait component increase of 40.8% for Positive Symptoms from baseline (53.9%) up to 9-months follow-up (98.4%) and an increase of 52.1% in Affectivity from baseline (31.1%) up to 6-month follow-up (83.2%). Negative Symptoms had the highest trait component of all subscales between baseline and 6-months follow-up, and the second highest apart from Positive Symptoms at 9-months and 12-months follow-up. *PPP*s ranged from 0.384 to 0.514 and upper levels of *CI* were all positive indicating a good fit of the latent state-trait models.

### Linear regression analysis

The examination of diagnostic residual plots, histograms and Q–Q-Plots revealed that the assumptions of linearity, homoscedasticity, independence and normality were met.

The results of the linear regression analysis comparing trait components of CHRS and FEP individuals are shown in Table 3.

**Table 3:** Linear regression analysis comparing trait components of CHRS and FEP individuals.

	β	*SD*	*p*-value	95% *CI* (β)		*PPP*	95% *CI* (Δχ^2^)	
Positive Symptoms	−0.024	0.003	< .001	−0.028	−0.018	0.247	−52.627	100.671
Negative Symptoms	−0.007	0.002	.001	−0.011	−0.003	0.440	−45.838	56.823
Affectivity	−0.028	0.002	< .001	−0.030	−0.024	0.219	−35.305	100.174
Resistance	−0.632	0.199	< .001	−0.963	−0.230	0.161	−23.374	86.464
Activation	−0.007	0.002	.001	−0.011	−0.003	0.504	−55.030	52.551
Excitement	−0.003	0.010	.436	−0.017	0.017	0.161	−43.091	107.946

*CHRS* Clinical High-Risk State, *FEP* First Episode Psychosis, *ß* standardized regression coefficient, *SD* standard deviation, *CI* confidence interval, *PPP* posterior predictive *p*-value, *Δχ*^*2*^ difference between the observed and the replicated chi-square values.

Subgroup analyses showed all subscales except Excitement to have lower scores on the trait component in CHRS than in FEP individuals. *PPP*s ranged from 0.161 to 0.504 and upper levels of *CI* were all positive indicating a good fit.

## Discussion

To the best of the authors’ knowledge, this is the first study to assess to what extent the psychopathological syndromes associated with CHRS and FEP are related to latent states and traits over a 1 year time-span.

The dimensions of psychopathological symptoms assessed with the BPRS differ in their latent state-trait structure: Whereas Positive Symptoms, Negative symptoms, Affectivity, and Activation show a relatively high trait component, Resistance and BPRS-EC present with low trait and relatively high state components. Negative Symptoms were the most consistent syndrome. Over all individuals, there is a development indicating an increasing proportion of trait components in psychosis syndromes over time. FEP individuals in general show a higher trait symptom severity than CHRS individuals with the exception of BPRS-EC.

The finding that Negative Symptoms have the highest trait component levels, is in line with previous literature discussing a predominant deficit component of schizophrenia and with clinical experience leading to a persistently impaired course of psychotic and negative symptoms throughout the illness^46^. For instance, a study of Strauss, et al.^46^ examining periods of recovery of individuals with and without deficit syndrome over 20 years found that individuals without a deficit syndrome showed more periods of global recovery than individuals with a deficit syndrome^47^. Another article of Millan, Fone, Steckler, and Horan (2014) gives an integrative overview of Negative Symptoms and discusses possible reasons for their persistence and treatment options^47^. The difference between CHRS and FEP individuals concerning the trait character of symptomatology is also consistent with the definition of these two groups: CHRS individuals are supposed to have more fluctuating symptoms while FEP individuals should show rather consolidated symptoms^19^. Furthermore, the finding of higher trait components in FEP than in CHRS individuals is compatible with van Os et al.^5^ postulating two concepts of psychosis, the psychosis continuum model and the psychosis-proneness-persistence-impairment model. Excitement and Resistance being more episodic supports the evidence from several studies showing that aggressive symptoms change over time as a function of the underlying illness and occur mostly during early treatment correlating with florid psychotic symptoms^48,49^. As Excitement showed the lowest trait component, this subscale should be used to monitor agitation and aggression in clinical practice.

The result that Positive Symptoms, Negative Symptoms, Affectivity, and Activation seem to be more persistent, while Excitement and Resistance seem to be more episodic, can be interpreted in different ways: On one hand, this finding supports the more common and deficit-oriented view that there may be some persisting symptom clusters, remaining after an acute psychotic episode as residual symptoms. Studies investigating community samples found that the positive symptoms delusional ideation and isolated hallucinations are relatively prevalent (15% to 16%) in the general population and pose a high risk for the development of a psychotic disorder^50–54^. In addition, negative symptoms can resist control even when positive symptoms are well treated^47^. The review of Lang et al. showed remission rates of around 50% and rather stable psychopathological symptom patterns with residual symptoms^55^. Following this line of thought, an adequate treatment of psychosis would mean to focus especially on these persevering symptom clusters.

On the other hand, the rather trait-characterized symptom clusters Positive Symptoms, Negative symptoms, Affectivity, and Activation can be seen as structural types of the disorder. This phenomenological approach, based on the Gestalt theory and described by Parnas^56^, considers that a psychosis symptom always appears in a holistic context and is part of a “whole” of mutually implicative, interpenetrating experiences, feelings, beliefs, expressions, and actions, influenced by the biographical background of the individual. Even if the conceptualization in the common diagnosis systems DSM-5 and ICD-10 is an important tool helping to communicate among healthcare professionals and ensuring a standardized documentation, it captures only a fragment of the clinical core of schizophrenia-spectrum disorders. As an example, the representatives name the drastically decreased number of cases diagnosed with hebephrenia, while the borderline personality disorder diagnosis increased since the ICD-10 was introduced, hinting on a degree of arbitrariness of the current operationalization of schizophrenia spectrum disorders^56^. Furthermore, there is evidence that psychopathology is not necessarily related to an impairment in subjective wellbeing: a study examining individuals with first episode psychosis showed no associations between subjective wellbeing and the PANSS subscales Negative Symptoms, Positive Symptoms, Disorganization and Excitement^24^, supporting a rather resource-oriented point of view and discouraging a perspective focused on residual symptoms. Following this line of thought, our results could provide a basis to disentangle the heterogeneity in the psychopathological presentation of schizophrenia-spectrum disorders and for the investigation of endophenotypes linked to risk factors and biological correlates^57^.

Strengths of the current post-hoc analysis include the relatively large sample size for this difficult-to-study sample and the equality of time between study visits, avoiding an inequality related bias.

Concerning the composition of the study sample, there are a few more aspects to discuss. The adequate sample size of both subsamples (CHRS, FEP) and the naturalistic nature of the study sample recruited from two established psychosis early detection and treatment programs constitute strengths of this study and enhance the generalizability of the results. On the other hand, only a limited common set of variables on socio-demographic and clinical parameters was available reducing the richness of detail of the sample description and limiting the possibilities of variables to control for (e.g. ethnicity, socio-economic status, intelligence quotient). Furthermore, data was extracted from a treatment as usual (TAU) population including psychopharmacological treatment in some cases. In addition, data on non-pharmacological treatment was unfortunately not available and could not be controlled for. Nevertheless, examining an untreated population would not have been possible due to ethical reasons, making a naturalistic design the best feasible solution. Additionally, there was a relatively high percentage of dropouts, what could have biased our results in the direction of underestimation of trait-components.

Another issue is the broad spectrum of diagnoses included in our analysis, especially the question if the findings could have been influenced by the inclusion of FEP individuals with schizoaffective psychosis. Due to the relatively small percentage of individuals with a schizoaffective psychosis (8.4%) and the similar trait component of Affectivity compared to the subscales Positive Symptoms, Negative symptoms, and Activation, we assume that this effect can be neglected.

Several further limitations of the current study have to be taken into account. The BPRS is based on observations of the individuals’ behaviour over the last fourteen days. As we assessed our patients every three months, potential symptom fluctuations between assessments could not be accounted for. In general, the BPRS and the PANSS focus on symptoms without taking psychosocial functioning into account what leads to a rather limited view of psychopathology. Further studies could integrate measures including the level of functioning to ensure a more comprehensive assessment of the latent state-trait structure of psychosis. In addition, psychopathology was measured using two different psychometric tools: CHRS individuals were assessed using the BPRS, and FEP individuals were assessed using the PANSS with post-hoc extraction of BPRS scores. However, the items of the BPRS and the PANSS have a large overlap and there is evidence showing their equivalence^20^. Furthermore, although psychopathology was assessed by trained raters in all cases, and although the FePsy and PEDIC programs are similar in their structure and have comparable inclusion and exclusion criteria, systematic differences depending on study centre cannot be completely ruled out, possibly influencing the comparison between CHRS and FEP data. Further monocentric studies are therefore needed to assess if differences found in the current analyses can also be replicated in a more homogeneous population.

Further studies should examine the latent state-trait structure of psychosis symptoms over a larger period of time and could include moderating factors like specific diagnoses (e.g. personality disorders).

To conclude, even if there are several methodological limitations, the current study contributes to a deeper understanding of psychosis and its symptom clusters. Additionally, our results are relevant for clinical practice: an increasing proportion of psychosis symptoms is persisting over time, underlining the importance of the early detection and treatment of psychosis symptoms. Furthermore, differentiating psychosis symptom clusters regarding their stability may help to set priorities, especially in the beginning of treatment.
